# Anti-Aging Implications of *Astragalus Membranaceus* (Huangqi): A Well-Known Chinese Tonic

**DOI:** 10.14336/AD.2017.0816

**Published:** 2017-12-01

**Authors:** Ping Liu, Haiping Zhao, Yumin Luo

**Affiliations:** ^1^Cerebrovascular Diseases Research Institute, and Department of Neurology, Xuanwu Hospital of Capital Medical University, Beijing, China; ^2^Center of Stroke, Beijing Institute for Brain Disorders, Beijing, China; ^3^Beijing Key Laboratory of Translational Medicine for Cerebrovascular Diseases, Beijing, China

**Keywords:** *Astragalus membranaceus*, aging, neurodegenerative disease, cancer, immnoregulation

## Abstract

Owing to a dramatic increase in average life expectancy and the Family Planning program of the 1970s - 1990s, China is rapidly becoming an aging society. Therefore, the investigation of healthspan-extending drugs becomes more urgent. *Astragalus membranaceus* (Huangqi) is a major medicinal herb that has been commonly used in many herbal formulations in the practice of traditional Chinese medicine (TCM) to treat a wide variety of diseases and body disorders, or marketed as life-prolonging extracts for human use in China, for more than 2000 years. The major components of *Astragalus membranaceus* are polysaccharides, flavonoids, and saponins. Pharmacological research indicates that the extract component of *Astragalus membranaceus* can increase telomerase activity, and has antioxidant, anti-inflammatory, immunoregulatory, anticancer, hypolipidemic, antihyperglycemic, hepatoprotective, expectorant, and diuretic effects. A proprietary extract of the dried root of *Astragalus membranaceus*, called TA-65, was associated with a significant age-reversal effect in the immune system. Our review focuses on the function and the underlying mechanisms of *Astragalus membranaceus* in lifespan extension, anti-vascular aging, anti-brain aging, and anti-cancer effects, based on experimental and clinical studies.

Owing to a dramatic increase in average life expectancy and the Family Planning program of the 1970s-1990s, China is rapidly becoming an aging society. Thus, aging and aging-associated diseases, such as neurodegeneration, cardiovascular disease, and cancer, are becoming some of the most important global problems. Aging is defined as a progressive decline in intrinsic physiological function, leading to an increase in age-specific mortality rate and a decrease in age-specific reproductive rate [1]. The major theories of aging include telomere shortening theory [2], epigenetic and genetic regulation theory [3,4], stem cell theory [5], mitochondrial dysfunction [6], metabolic and immune deregulation [7,8], proteostasis loss [9], and gut microbiota regulating theory [10]. Hence, targeting these pathological changes could reverse aging and treat age-associated diseases. In traditional Chinese medicine, some herbals have been used for anti-aging since ancient times.

*Astragalus membranaceus* (Huangqi), as one of the most important Qi tonifying adaptogenic herbs in Trational Chinese Medicine, has a long history of medicinal use. *Astragalus membranaceus* was originally described in the Shennong's Classic of Materia Medica, the earliest complete Pharmacopoeia of China written from the Warring States Period to Han Dynasty [11,12]. It is valued for its ability to strengthen the primary energy of the body which we know as the immune system, as well as the metabolic, respiratory and eliminative functions. This fact is being increasingly substantiated by pharmacological studies showing that it can increase telomerase activity, and has antioxidant, anti-inflammatory, immune-regulatory, anticancer, hypolipidemic, antihyperglycemic, hepatoprotective, expectorant, and diuretic effects [13-18]. Specifically, constituents of the dried roots of Astragalus spp. Radix Astragali provide significant protection against heart, brain, kidney, intestine, liver and lung injury in various models of oxidative stress-related disease [19,20]. In order to clarify the potential application of *Astragalus membranaceus* in anti-aging, we summarize the effect and mechanism of its extracts and effective component monomer against aging and age-related disease. This information could help clinicians and scientists develop novel target-specific and effective therapeutic agents that are deprived of major systemic side effects, so as to establish a better treatment regimen in the battle against aging.

## 1. Phytochemistry of *Astragalus membranaceus*

*Astragalus membranaceus* includes over 2000 species, among them, *Astragalus membranaceus* (Fisch.) Bge. (Fam Leguminosae) and *Astragalus membranaceus* (Fisch.) Bge. Var. mongholicus (Bge.) Hsiao are the most commonly used. In traditional Chinese medicine, *Astragali Radix*, the root of* Astragalus membranaceus*, was used in patients with chronic diseases and healthy persons who want to improve their body vital functions.

At present, more than 200 compounds have been isolated and identified from *Astragalus membranaceus*. Its total polysaccharides, saponins and flavonoids fractions and several isolated compounds have been the most studied, and these bear the biological activities of *Astragalus membranaceus* [21,22].

### 1.1 Triterpenoid Saponins (Astragalosides)

There are more than 161 saponins including about 142 kinds of cycloartane-type saponins and about 19 kinds of oleanane-type saponins. Among them, 5 major saponins, including astragalosides I, II, and IV, and isoastragaloside I and II, all being cycloartanetype triterpenoids, make up more than 80% of the total saponins. And Astragaloside IV is the qualitative control biomarker [23].

### 1.2 Flavonoids

There are more than 63 flavonoids mainly including isoflavones, isoflavans, pterocarpans, flavonols, flavones, and flavonones. Among them, isoflavones are the major constituents, and calycosin-7-O-β-D-glucoside, as one of the isoflavones, is the dominant component, and used as a chemical marker in quality analyses of *Astragalus membranaceus* [24]. In addition, there are 3 special flavonoids including sulfuretin, isoliquiritigenin, and pendulone.

### 1.3 Polysaccharides

Knowledge about the precise chemistry of Astragalus polysaccharides is quite limited. Since polysaccharides are macromolecules with complicated chemical structures, it is relatively difficult to isolate and characterize their individual components. There are 14 polysaccharides in *Astragalus membranaceus*, and 13 kinds of them have β-D-(1→3)-galactan moieties branched with β-D-(1→6)-galactooligosaccharide side-chains[25,26].

## 2. Anti-aging and anti-aging-related effects of extracts of *Astragalus membranaceus*

### 2.1 Oxidative stress reduction by extracts of *Astragalus membranaceus*

*Astragalus membranaceus* can inhibit the oxidant stress by up-regulating the antioxidant factors. Aqueous extract of *Astragali radix* decreased the myocardial infarction size and improved the cardiac function in a myocardial ischemic rat model, which is related to the antioxidant effects via maintaining the activity of superoxide dismutase (SOD), decreasing the production of malondialdehyde (MDA) and free radical levels, and reducing cell apoptosis [27]. Besides, Astragalus injection can decrease the reactive oxygen species (ROS) and MDA levels in a rat model of cerebral ischemia through up-regulating the expression of nuclear factor erythroid 2-related factor 2 (Nrf-2), SOD, catalase and glutathione peroxidase (GSH-Px) [28].

### 2.2 Immunomodulatory effects by extracts of *Astragalus membranaceus*

*Astragalus membranaceus* was used to promote immune function and as a tonic to build the stamina [29]. The aqueous extract of *Astragali radix* also has significant immunological adjuvant activity when compounded with human vaccines [30].

*Astragalus membranaceus* could affect the innate immune response. The aqueous extract of *Astragali radix* induced the activation and migration, and monocyte maturation of peripheral blood mononuclear cells [31]. In the macrophage cell line RAW 264.7, aqueous extract of *Astragali radix* reversed the increasing iNOS expression and NO production by lipopolysaccharide (LPS), and reduced the suppression of macrophage cell proliferation by methotrexate [32]. Another research found that in the macrophage cell line ANA-1, aqueous extract of *Astragali radix* inhibited cytokine production via depressing p38 MAPK and NF-κB signaling pathways induced by advanced glycation endproduct [33].

*Astragalus membranaceus* could also affect the acquired immune response. The aqueous extract of *Astragali radix* exhibits mitogenic activities on T-cell depleted populations, augments the antibody response, and restores the lymphocyte blastogenic response in aging mice [34]. And aqueous extract of *Astragali radix* activated CD4^+^ and CD8^+^ T cells of humans without influencing proliferation [35,36]. In addition, the ethanol extracts of *Astragali Radix* selectively alter Th1/Th2 cytokine secretion patterns of CD4^+^ T cells through enhancing the IL-4 and IL-10 levels in Th2 cells and reducing the levels of IL-2 and IFN-γ in Th1 cells [37,38]. Similarly, hydroalcoholic extract of *Astragalus gypsicolus* also modulated the balance of Th1/Th2 cytokines in allergic mice model [39].

Beyond that, aqueous extracts of *Astragali radix* promote myelopoiesis in myelosuppressed mice by improving the hematopoietic microenviroment, including enhancing the survival of bone-marrow-derived mesenchymal stem cells (BMSCs), proliferation of colony-forming unit-ﬁbroblast through upregulation of granulocyte-macrophage colony stimulating factor and of bcl-2 expression in BMSCs [40].

### 2.3 Anti-neurodegeneration by extracts of *Astragalus membranaceus*

The aqueous extract of *Astragali radix* can reverse the memory impairment and neurodegeneration. It can increase the number of M-cholinergic receptors in the cortex, hippocampus and striate body of a senile rat model [41]. Moreover, it can prevent the loss of axons and synapses in the cortex and hippocampus, and reverse the memory loss of amyloid β-peptide (Aβ)-induced cognitive deficits mice [42].

### 2.4 Anti-tumor effect by extracts of *Astragalus membranaceus*

The extract of *Astragali radix,* alone or combined with chemotherapeutic agents, has positive effects on the treatment of many tumors [43].

The aqueous extract of *Astragali radix* could inhibit the proliferation and invasion of digestive tumor cells in a dose-dependent manner. In hepatocellular carcinoma cell (HCC) line HepG2, the aqueous extract of *Astragali radix* combined with Salvia miltiorrhiza inhibited the invasion of tumor cells by modulating TGF-beta/Smad signaling [44]. Further research found that in the HCC rat model induced by diethylinitrosamine (DEN), the aqueous extract of *Astragali radix* combined with Salvia miltiorrhiza reduced the incidence and multiplicity of HCC development in a dose-dependent manner by inhibiting fibrosis and the transcription of plasminogen activator inhibitor-1 [45,46]. In addition, the aqueous extract of *Astragali radix* can inhibit the growth of human gastric cancer cell lines AGS and KATO-III in a time-and dose-dependent manner [47]. It was further found that Astragalus injection can reduce the apoptosis by increasing the Bcl-2 expression and reducing the Bax expression in gastric cancer cell supernatant-induced peritoneal mesothelial cells [48].

Extract of* Astragali radix* also has anti-tumor effects against lung cancer, renal cell carcinoma, and bladder cancer, as shown in clinical trials and animal experiments. The Astragalus-based traditional Chinese medicine formula increased the effectiveness and reduced the toxicity of standard platinum-based chemotherapy in 2,815 patients with advanced non-small-cell lung cancer [49]. In addition, aqueous extracts of *Astragali radix* may have exerted their antitumor effects via augmentation of phagocyte and LAK cell activities in mice bearing renal cell carcinoma [50]. Similarly, aqueous extract of *Astragali radix* lowered the incidence of urinary bladder carcinoma through activating the cytotoxicity of lymphocytes and increasing the production of IL-2 and IFN-γ in mice [51]. Therefore, immune regulation is one of its main mechanisms of anticancer protection.

## 3. Anti-aging and anti-aging-related effects of *Astragalus membranaceus* saponins (AMS)

### 3.1 Total AMS

#### 3.1.1 Immunomodulatory effects of total AMS

AMS not only regulate the activity of immune cells, but also regulate the expression of adhesion molecules on the surface of endothelial cells. AMS can suppress LPS-induced iNOS and TNF-α expression in the mouse macrophage RAW264.7 via suppressing p38 MAPK/NF-κB signaling [52]. In primary mouse kidney arterial endothelial cells, total AMS attenuated TNFα-induced up-regulation of cell adhesion molecules, activation of NF-κB, degradation of inhibitor of κBα (IκBα), and inhibited pro-apoptotic signaling pathway by reducing cell surface TNFR1 level [53].

#### 3.1.2 Anti-tumor by total AMS

The anti-cancer effects of total AMS have been investigated in digestive system tumors [53-58].

AMS can inhibit the proliferation and induce apoptosis of colon cancer. AMS inhibited cell proliferation by accumulation in S phase and G2/M arrest and promoted apoptosis in human colon cancer cells HT-29. It targeted NSAID-activated gene, causing overexpression of nonsteroidal anti-inflammatory drug activated gene-1 (NAG-1) and the caspase activation through modulation of an ERK independent NF-κB signaling pathway [56,59]. AMS also exerted anti-carcinogenic activity through modulating the PI3K/Akt/mTOR and ERK signaling pathways in colon cancer cells HCT 116 and HT-29 [57,60]. Another study found AMS could induce endoplasmic reticulum (ER) stress-mediated apoptosis by elevating the level of XBP-1 and CCAAT/enhancer-binding protein-homologous protein [61].

In human gastric adenocarcinoma cells, AMS arrested cell cycle at the G2/M phase through regulating cyclin B1, p21 and c-myc [58]. Besides its pro-apoptotic and anti-proliferative activities, AMS can modulate the invasiveness and angiogenesis of tumor cells, by down-regulating the VEGF and metastatic proteins metalloproteinase (MMP)-2 and MMP-9, which suggested that AMS may be used for the treatment of advanced and metastatic cancers [58].

### 3.2 Astragalosides (AST)

AST are cycloartane triterpenoid saponins which are characterized by 3-, 6- and/or 25-conjugated glucose moieties and whose 3-glucose is acetylated.

#### 3.2.1 Anti-aging effect of AST

AST delay the senility in the aging rat induced by hydrocortisone through the antioxidant and immunoregulatory effects [62]. Moreover, treatment with AST for 10 weeks could delay senility and ameliorate age-related alternations in both motor response and memory in the D-galactose-induced senescent mouse and the pre-aged (17-month-old) mouse [63]. The anti-aging mechanism was suggested to act by restoring the activities of MnSOD and GSH/GSSG ratio, as well as enhancing the thymus index and splenocytes proliferation [63]. AST also protected human skin fibroblasts against ultraviolet A (UVA)-induced photoaging, reducing the senescence-associated β-galactosidase positive cell rate via decreasing the level of MMP-1 and increasing the expression of tissue inhibitor of metalloproteinase-1 and transforming growth factor-β1 (TGF-β1) [64].

#### 3.2.2 Immunomodulatory effects of AST

AST promote both humoral and cellular immune responses of chicken vaccinated with Newcastle disease [65]. Also, AST increased CD45 phosphatase activity in mice primary splenocytes and T cells [66].

#### 3.2.3 Anti-vascular disease by AST

AST have positive effects on cardio-cerebrovascular diseases. AST promoted angiogenesis through increasing the expression of VEGF and bFGF in the rat myocardial infarction model [67]. AST also improved left ventricle function and cardiac structure of chronic heart failure rats through reversing the depression of sarcoplasmic reticulum Ca²?-ATPase activity, and increasing phosphorylated phospholamban, similar with the result of previous studies in cultured cardiac myocytes of neonatal rats [68,69]. In the rat model of focal cerebral ischemia-reperfusion injury, AST protected against neurological deficit by regulating the expressions of iNOS, nerve growth factor and tropomyosin receptor kinase A [70]. Further research found that AST blocked apoptosis in the PC12 cells of OGD model by suppressing functional impairment of mitochondria and ER stress through regulating the P38 MAPK signaling pathway [71].

#### 3.2.4 Anti-neurodegeneration by AST

AST improved learning and memory in the AD model of 12 month old male rats through down-regulating the expression of APP, β-secretase and Aβ(1-40) in hippocampus [72]. In addition, AST attenuated Aβ25-35-induced cytotoxicity degeneration of primary rat cortical cells, inhibited apoptosis, synaptotoxicity, and mitochondrial dysfunction via modulation of both PI3K/AKT and ERK pathways [73]. In addition, AST also improved the learning and memory abilities in a cerebral ischemia rat model by attenuating neuronal cells apoptosis though increasing the expression of p-ERK and p-Akt, decreasing the expression of p-JNK [74].

#### 3.2.5 Anti-tumor effects by AST

There is little research done concerning the effects of AST on tumors. Yet, it was shown that AST pronouncedly inhibited the proliferation of human gastric cancer MKN-74 cells in a dose and time-dependent manner [75].

### 3.3 Astragaloside II (AST-II)

AST-II is a principal component of astragalosides purified from *Radix Astragali*. Its chemical structure is (3b,6a,16b,20R,24S)-3-((2-O-Acetyl-betaD-xylopy-xylopyranosyl)oxy)-20,24 -epoxy-16,25-dihydroxy-9,19-cyclolanostan-6-ylbeta-D-glucopyranoside), and its molecular formula is C45H72O16, which can regulate the immune response and inhibit tumor.

#### 3.3.1 Immunomodulatory effects of AST-II

In both cyclophosphamide-induced immunosuppressed mice and primary splenocytes and T cells, AST-II promoted ConA- and alloantigen-induced T cell proliferation and activation via regulating the activity of CD45 PTPase and initiating the activation cascade via CD45-mediated dephosphorylation of the Lck tyrosine kinase [66]. But AST-II has no obvious effects on LPS-induced B cell proliferation. Although AST-II is an immunological adjuvant, it is the most toxic Astragalus component with 5-10 % weight loss in mice [76].

#### 3.3.2 Anti-tumor by AST-II

AST-II is a multidrug resistance (MDR) reversal agent and a potential adjunctive agent for hepatic cancer chemotherapy. In the human hepatic cancer Bel-7402 and Bel-7402/FU cells, AST-II downregulated the expression of mdr1 gene and P-glycoprotein, suppressed autophagy by interfering with Beclin-1 and LC3 via MAPK-mTOR pathway, through which it sensitized human cancer resistant cells to 5-FU-induced cell death [77,78].

### 3.4 Astragaloside IV (AST-IV)

AST-IV is a principal component of astragalosides. Chemical structure of AS-IV is 3-O-beta-D-xylo-pyranosyl-6-O-beta-D-glucopyranosyl-cycloastragenol, a lanolin alcohol-shaped tetracyclic triterpenoid saponin with high polarity, and its molecular formula is C14H68O14. In beagle dogs, the absolute bioavailability of AST-IV by oral is only 7.4%, and its plasma protein binding rate is about 90% [79]. And the reasons include its poor intestinal permeability, high molecular weight, low lipophilicity and its paracelluar transport [80].

#### 3.4.1 Anti-aging effect of AST-IV

AST-IV is an effective agent for the prevention of photoaging. It enhances the viability of skin cells irradiated with UVA, suppresses the expression of MMP-1 and prevents the degradation of type I procollagen through suppression of MAPK and NF-κB pathway and regulating TGF-β/Smad pathway [81,82].

#### 3.4.2 Immunomodulatory effects of AST-IV

AST-IV enhances immune function as an immunological adjuvant with no toxicity and has anti-inflammatory effect [76]. AST-IV increased the proliferation of T and B lymphocytes and antibody production but inhibits the production of IL-1 and TNF-α from peritoneal macrophages [83]. AST-IV inhibits the function of high-mobility group box 1, and shifts of Th2 to Th1 through upregulating Foxp3 expression on CD4^+^CD25^+^ regulatory T cells (Tregs) [84,85]. AST-IV and cycloastragenol could upregulate telomerase activity, improve the proliferation and enhance immune function of CD8+ T lymphocytes from HIV-infected patients [86], through activating Src/MEK/ERK pathway in a time- and dose-dependent manner [87].

AST-IV also has anti-inflammatory activity in endothelial cells. AST-IV significantly inhibited LPS- and TNFα-induced expression of E-selectin and VCAM-1 through inhibiting the NF-κB pathway [88].

#### 3.4.3 AST-IV effects on vascular-related diseases

In acute myocardial infarction rat model, AST-IV preserved the cardiac function, decreased the infarcted size and inhibited the left ventricular fibrosis [89]. AST-IV decreased the ROS and MDA levels by upregulating the content and activity of GSH-Px and SOD in neonatal rat primary cardiomyocytes [90,91]. AST-IV can attenuate the inflammatory cytokines by down-regulating the TLR4/NF-кB pathway, up-regulating Sonic hedgehog pathway and the TGF-β expression [92,93].

In the murine cerebral ischemia model, AST-IV reduced infarct volume, and improved neurological outcome by decreasing the expression of peripheral benzodiazepine receptors [94]. AST-IV down-regulated the MDA content and ROS production, restores the activity of SOD and GSH-PX [95,96]. AST-IV alone or combined with ginsenoside Rg1, ginsenoside Rb1 and notoginsenoside R1, strengthened the antagonistic effects on oxidative stress injury through activating Nrf2/HO-1 pathway [97,98]. In addition, AST-IV prevented neutrophils accumulation in the brain parenchyma, and downregulated inflammatory reaction by inhibiting NF-κB [99].

AST-IV has the angiogenic effect in pathological conditions. In human umbilical vein, endothelial cells (HUVECs) exposed to hypoxia, AST-IV promoted cell proliferation and migration, and increased tube formation [100]. It might be that AST-IV enhanced the expression of VEGF and kinase domain region/fetal liver kinase-1/VEGF receptor 2 (KDR/Flk-1/VEGFR2) [101]. And pretreatment with AST-IV in H_2_O_2_-induced HUVECs showed antioxidant properties via inhibiting NADPH oxidase-ROS-NF-κB pathway [102]. However, some other study found that in vascular human dermal vascular smooth muscle cells induced by platelet-derived growth factor, AST-IV inhibits the proliferation and migration by down-regulating MMP2 and the p38 MAPK signaling pathway [103]. These contradictory results may be due to their use of different cells, and this issue needs further research in vitro and in vivo.

#### 3.4.4 AST-IV protective effects against neurodegeneration and cognitive decline

In the AD mouse model, AST-IV could improve the learning and memory deficits, inhibit Aβ plaque accumulation in the brain of APP/PS1 mice [104]. AST-IV prevents Aβ1-42-induced neurotoxicity and increases cell viability of SK-N-SH cells. This may be because AST-IV maintained the function of mitochondria such as increasing ΔΨm, the ATP level and the cytochrome c oxidase activity through inhibiting mPTP opening, inhibited the mitochondria-mediated apoptotic process [105]. Both* in vivo* and *in vitro*, AST-IV treatment inhibits the expression of β-site amyloid precursor protein cleaving enzyme 1 (BACE1) by activating peroxisome proliferator-activated receptor gamma (PPARγ) [104]. AST-IV also attenuated memory impairment induced by bilateral common carotid artery occlusion in mice. It attenuated neuronal apoptosis, suppressed oxidative damage, reduced the over activation of microglia and astrocyteы in the hippocampus, down-regulated the NLRP3 inflammasome overactivation by reducing the expression of toll-like receptor-4 (TLR4) and the phosphorylation of NF-κB [106,107]. The neural stem cell (NSC) transplantation is a potential therapeutic approach for AD. In an AD model transplanted with NSC into the hippocampus, AST-IV increased the proliferation of NSC and immature neurons and promoted the learning and memory by up-regulating Notch-1 and down-regulating presenilin-1 (PS-1) [108].

AST-IV may reverse behavioral deficits associated with PD. In the in vitro PD model of primary nigral neuron cells induced by 6-hydroxydopamine (6-OHDA), AST-IV attenuated the loss and degeneration of dopaminergic neurons, increased the number of tyrosine hydrolase immunopositive neurons, and increased the NOS level of dopaminergic neurons [109]. In the in vitro PD model of SH-SY5Y cells induced by 1-methyl-4-phenylpyridnium ion (MPP(+)), pretreatment with AST-IV attenuated the cell death and increased the cell viability, by down-regulating the Bax/Bcl-2 ratio and the activation of cleaved caspase-3 [110].

In addition, one of the therapeutic targets for neurodegenerative diseases is inhibiting microglia activation. AST-IV can inhibit microglia activation by activating the glucocorticoid pathway, suggesting its possible therapeutic potential [111].

#### 3.4.5 Anti-tumor effects by AST-IV

AST-IV reduced the proliferation, migration and invasion of several tumors.

In HCC cell lines HepG2, AST-IV inhibited the colonogenic survival and anchorage-independent growth of cancer cells, down-regulated the expression of oncogene Vav3.1 in a dose- and time-dependent manner [112]. AST-IV also attenuated the migration and invasion of Huh7 and MHCC97-H cell line through inhibiting the epithelial mesenchymal transition by targeting the Akt/GSK-3β/β-catenin pathway [113]. Moreover, in orthotopic HCC mouse model, AST-IV significantly reduced the tumor weight, decreased the count of tumor microvessels and the expression of angiogenic factors including MMP2, FGF2, VEGF, and HGF, which might be due to up-regulating a tumor suppressor gene miR-122, while down-regulating an oncogene miR-221 [114].

In a lung cancer mice model induced by indoleamine 2,3-dioxygenase, AST-IV suppressed the tumor growth, interfered with T-cell immunity by decreasing Tregs and increasing cytotoxic T lymphocytes among the splenetic mononuclear cells [115]. In vitro, AST-IV inhibited the migration and invasion of lung cancer A549 cells, regulated the expression of MMP-2, MMP-9 and decreased the inflammation factors TNF-α and IL-6 through downregulating the PKC-α-ERK1/2-NF-κB pathway [116]. In breast cancer cell line MDA-MB-231, AST-IV inhibited the viability and invasion of tumor cells, downregulated the expression of Vav3, MMP-2, and MMP-9 through suppressing the activation of ERK1/2 and JNK [117].

AST-IV enhances the chemosensitivity of chemotherapeutic drugs in cancers. In colorectal cancer, AST-IV inhibits the growth of cancer cells with no cytotoxicity in normal colonic cells, and increases the chemosensitivity of cancer cells to cisplatin through inhibition of NOTCH3 [118]. In human non-small cell lung cancer cell lines, high doses of AST-IV inhibited cell growth, and increased chemosensitivity to cisplatin by down-regulating the mRNA and protein levels of B7-H3 [119].

In summary, AST-IV exerts anti-tumor effects; it may be a stand-alone alternative therapy for tumors with no side effects, or a part of combined therapy together with chemotherapeutic drugs.

## 4. Anti-aging and anti-aging related effects of Astragalus flavonoids (AF)

Flavonoids are the largest group of polyphenolic compounds of *Astragalus membtanaceus*. They include flavones, flavonols, flavanones, flavanonols, chalcones, aurones, isoflavonoids and pterocarpans. Among them, isoflavonoids are divided into two groups: isoflavones and isoflavanes.

### 4.1 Immunomodulatory effects of AF

Oral treatment with AF for 6 weeks enhances immune function through ameliorating the reduced spleen cell proliferation and balancing the abnormal cytokine levels in rats with induced chronic fatigue syndrome [120]. In addition, AF promotes the proliferation and activation of natural killer cell line NK-92, upregulates the expressions of activating receptors NKG2D, NKp44 [121]. AF also has significant anti-inflammatory effects and immunological adjuvant activity [76]. AF inhibited the production of the proinflammatory cytokines IL-6 and IL-12 p40 in bone marrow-derived dendritic cells (DCs) stimulated by LPS [122]. And AF showed anti-NO activity in mouse macrophage RAW264.7 cells stimulated by LPS [123]. Further study indicated that formononetin, one of isoflavonoids, significantly inhibited the production of NO and the expression of iNOS and cyclooxygenase-2 (COX-2) in LPS-stimulated RAW 264.7 cells [124,125].

### 4.2 Oxidative stress reduction by AF

In the atherosclerotic rabbit model induced with high cholesterol feeding, AF reduced the plasma levels of total cholesterol and LDL-cholesterol, increased the level of HDL-cholesterol, scavenged superoxide and hydroxyl radicals, and reduced the aortic fatty streak area [126]. In the hemorrhagic shock/reperfusion injury rabbit model, AF had a protective effect on ischemia/reperfusion injury by blocking the decrease of NO and maintaining the acid-base balance of the body [127].

Isoflavonoids, including formononetin, calycosin and calycosin 7-O-glc, have neuronal protections in a PC12 cell model by scavenging free radicals generated by DPPH in a dose-dependent manner [128]. The antioxidant activity of isoflavonoids include preventing the decrease in the activity of antioxidant enzymes, decreasing membrane fluidity to stabilize membranes, chelating iron and copper ions involved in free radical production, scavenging ROS [128-131].

### 4.3 Anti-neurodegeneration and cognitive decline by AF

In vitro, neurodegenerative disorder model is induced by N-methyl-D-asparate (NMDA) in primary-cultured cortical neurons. As a novel herbal isoflavonoid isolated from *Astragalus membranaceus*, formononetin protects neurons and attenuates the apoptotic cells by increasing the levels of Bcl-2 and pro-caspase-3 and decreasing the levels of Bax and caspase-3 [132].

### 4.4 Anti-tumor effects by AF

AF can be used to treat many tumors, due to their effects of inhibiting the proliferation and metastasis of tumor cells, and apoptosis induction.

In human hepatocarcinoma cells SMMC-7721 and HepG2, AF inhibited the growth of tumor cells by arresting cell cycle in the G0/G1 and S phases, inducing apoptosis via mitochondria-dependent and death receptor-dependent apoptotic pathways [133]. In the hepatic cancer rat model induced by N-diethylnitrosamine, a flavonol glycoside rhamnocitrin 4′-β-d-galactopyranoside can prevent hepatocellular carcinogenesis by increasing the activity of antioxidant enzymes, such as SOD, CAT, GPx, GST and reducing the level of lipid peroxidation [134,135].

In human colon cancer, AF has pro-apoptotic and anti-tumorigenic activities, and elicits NAG-1 overexpression, a divergent member of the TGF-β superfamily which is correlated with growth inhibition and apoptosis induction [136,137]. Apigenin and quercetin, two flavone aglycones isolated from *Astragalus verrucosus Moris* have dose-dependent cytotoxic effects on human colon carcinoma cell line HCT116 [138]. Formononetin, another active compound, inhibits the cell growth and promotes apoptosis by caspase activation and downregulation of the Bcl-2 and Bcl-xL in HCT116 cells [139].

In nude mice inoculated with breast cancer cell line, AF prolongs the survival time, and inhibits the proliferation and metastasis of tumor cells in a dose-dependent manner [140]. AF and an isoflavone aglycone calycosin inhibit the proliferation through arresting cell cycle in the G0/G1 phase by decreasing cyclin D1 in human erythroleukemia cell line K562 [141]. Another flavonol glycoside, rhamnocitrin 4’-D-galactopyranoside, indicates the anti-proliferative effect in T-cell leukemia cells SKW-3 [142].

## 5. Anti-aging and anti-aging-related effects of Astragalus polysaccharides (APS)

### 5.1 Total APS

#### 5.1.1 Anti-aging effects by APS

APS have anti-aging activity. In Caenorhabditis elegans, APS can extend healthy lifespan by up-regulating the miR-124 which targets an endoplasmic reticulum stress-regulated transmembrane transcription factor ATF-6 [143]. In male BALB/c mouse and D-galactose-induced aging mouse, APS increased the activity of SOD, GSH-Px, and CAT, decreased MDA production, and upregulated the thymus index and the spleen index [144,145].

#### 5.1.2 Antioxidant stress by APS

APS shows both in vitro and in vivo antioxidant activities [146,147]. APS increases the levels of SOD, glutathione and total antioxidant capacity and anti-hydroxyl radical activities, inhibiting the MDA formation [148]. APS inhibits the mitochondrial permeability transition, protects mitochondria from oxidative damage, and increases the activities of the antioxidases in mitochondria of mouse liver and brain [145].

#### 5.1.3 Immunoregulation by APS

APS possess an excellent immunopotentiatory property in both humoral immunity and cellular immunity [149]. In the common carp, APS up-regulates the gene expressions of IL-1β, TNF-α and lysozyme-C in the kidney, gill and spleen in a dose-dependent manner [150]. APS could also enhance the expression of IL-2, IL-3, IL-4, IFN-γ, IgM and IgG but decrease IgE [151-153].

Dendritic cells (DC) are special antigen presenting cells, and can initiate the primary immune response. APS prompts DCs maturation and their ability of antigen presentation and reduces the endocytic activity of DCs [154]. APS could also induce the differentiation of DCs and subsequently activate T cells [155]. APS can regulate the T cell immunity. APS can inhibit the activity of Tregs through binding anti-TLR4 on Tregs, and trigger a shift from Th2 to Th1 with activation of CD^4+^ T cells [156,157]. Besides, APS can activate B lymphocytes via membrane Ig in a TLR4-independent manner [158].

APS can activate mouse macrophages through triggering TLR4-mediated signaling pathways which upregulate the expression of p-p38, p-ERK, p-JNK, induce IκB-α degradation and NF-κB translocation, then finally enhance the production of TNF-α, IL-6, and NO [158,159]. APS also increases the level of granulocyte-macrophage colony-stimulating factor in mouse macrophages by activation of NF-κB/Rel [160,161]. APS can increase the number of mouse peritoneal macrophages and the deposition of the C3 [162]. APS also increases Ca^2+^-cAMP and TLR4/NF-κB signaling pathways in RAW 264.7 cells [163]. By contrast, APS suppress the expression of TNF-α and IL-1β by inhibiting NF-κB activation in the THP-1 macrophages stimulated by LPS [164]. And APS can ameliorate palmitate-induced pro-inflammatory responses through up-regulating the anti-inflammatory genes expression and down-regulating the pro-inflammatory genes expression via the AMPK signaling pathway [165].

APS is an immune adjuvant and can help vaccine to provide a longer-term protection. APS enhances the immune responses when combined with hepatitis B virus DNA vaccine or recombinant hepatitis B surface antigen vaccine. APS could resist the immunosuppression in a chicken model induced by cyclophosphamide through promoting T-lymphocyte proliferation and raising the serum levels of antibody titers [152]. In chickens vaccinated with Newcastle disease, APS liposome promotes the lymphocyte proliferation, and enhances antibody titer [166]. In an vivo model immunized with foot-and-mouth disease virus vaccine, APS was able to upregulate both the cellular and humoral immune response via increasing the phagocytic capacity of peritoneal macrophages, DCs maturation, T-lymphocyte proliferation, expression of cytokines and antibody production, enhancing the number of T helper memory cells, cytotoxic T cells, and natural killer cells among peripheral blood lymphocytes [167,168].

#### 5.1.4 APS protective effects against vascular diseases

APS inhibits apoptosis, and adhesion function damage in TNF-α-treated HUVECs through downregulating the expression of ICAM-1 and VCAM-1 via suppressing NF-κB activation [169]. APS inhibits the inflammatory response of rat bone marrow endothelial progenitor cells (EPCs) induced by thrombin, suppresses the expression of ICAM-1 by blocking NF-κB signaling and up-regulating expression of VEGF and its receptors Flt-1, KDR [170]. APS inhibits the cell apoptosis of human cardiac microvascular endothelial cells induced by Na_2_S_2_O_4_ via enhancing the levels of SOD, Bcl-2, PI3K/AKT, reducing the levels of ROS, Ca^2+^, MDA and Bax, and inhibiting the activity of caspase-3 [171]. In the foam cells, APS promotes the expression of ATP-binding cassette transporter A1, increases cholesterol effluent rate, and attenuates NF-κB nuclear translocation induced by TNF-α [172]. And in high-fat diet mouse, APS reduces plasma cholesterol by up-regulating the expression of cholesterol metabolism associated gene cholesterol-7α-hydroxylase and LDL-receptor, increasing fecal bile acid and neutral sterol excretion and inhibiting intestinal fractional cholesterol absorption [173]. All of these indicate that APS may have antiatherosclerotic activity. In addition, APS could promote angiogenesis. In hind limb ischemia rats, APS administered intramuscularly could increase the expression of VEGF, VEGFR-1, VEGFR-2, Ang-1 and Tie-2 [174].

#### 5.1.5 Prevention of neurodegeneration and cognitive decline by APS

In *C. elegans* and cell models, APS reduces polyglutamine aggregation and alleviates the neurotoxicity through the DAF-16/FOXO transcription factor which is known to regulate the lifespan [175]. APS can reverse the memory impairment in the diabetic model, and the potential mechanisms are associated with their antioxidant properties, influence on insulin resistance and the regulation of lipid and glucose metabolism [176]. APS attenuates the inflammatory responses of BV2 induced by LPS, for example, reduces the production of IL-1b, TNF-α, and NO, inhibits COX-2 gene expression, via suppression of NF-κB signaling pathways [177].

#### 5.1.6 Anti-tumor effects by APS

APS exhibit anti-tumor activities, such as inhibiting proliferation, promoting apoptosis, regulating immune response.

APS can improve the quality of life of patients with digestive system cancer. In patients with esophageal cancer, APS combined with radiotherapy can improve the quality of life and reduce the incidence of nausea and leucopenia [178]. Meta-analysis indicated that in patients with gastric cancer, APS combined with chemotherapy can strengthen the most the overall response rate, improve the quality of life, reduce the incidence of nausea, vomiting and leukopenia [179]. APS can stimulate immunity activities in rats with gastric cancer by increasing the proliferation of spleen lymphocytes, the number of CD^4+^/CD^8+^ T cells, NK activities, and the levels of IL-2, IgA, IgG and IgM [180]. In addition, APS inhibit the proliferation of HepG2 cell line, induce apoptosis and arrest cell cycle in the G1 and sub G1/apoptotic phases [181,182]. Consistent with this, APS decrease cell viability and induce the apoptosis of H22 cells through regulating the expression of Bcl-2 and BAX, and of caspase by inhibiting Notch1 expression [183]. Moreover, APS enhance the chemosensitivity in H22 cells resistant to Adriamycin by downregulating the reversal of mdr1 expression and inhibiting the P-glycoprotein efflux pump function [184]. In H22-bearing mice, APS produce a tumor inhibition rate of 59.01%, increase the spleen and thymus indexes, and promote cell apoptosis by increasing Bax and decreasing Bcl-2 expression, improve the phagocytotic function of macrophages [185,186]. In the human HCC tissue, APS can restore the cytokine balance, decrease the population of CD4^+^CD25^high^ Treg cells and their proliferation by downregulating FOXP3 expression, and reduce the migration by inhibiting the secretion of the chemokine SDF-1 in a dose- and time-dependent manner [187]. APS also improve proliferation and activity of intestinal intraepithelial γδT cells in tumor-bearing mice by increasing the levels of IFN-γ, FasL and GrB in γδT cells [188]. In addition, APS inhibit the tumor cell growth in Kunming mice with Ehrlich's ascites carcinoma, decrease Bcl-2 and CDK4 levels, increase the percentage of CD^3+^ and CD^4+^ T-lymphocytes, the ratio of CD^4+^/CD^8+^ T cells and the expression of IL-2/IL-2R in spleen and Bax in tumor tissue [189].

APS can also improve the quality of life of patients with respiratory system tumor. In the nasopharyngeal carcinoma cell lines and in the xenograft model, APS promoted the anti-proliferative and apoptotic effects of cisplatin in a dose-dependent manner by upregulating the Bax/Bcl-2 ratio and increasing the expression of caspase [190]. In 136 patients with advanced NSCLC, APS injection integrated with vinorelbine and cisplatin, was able to decrease the fatigue, nausea and vomiting, pain and loss of appetite. However, there was no statistically significant change in objective response rate, median survival time and 1-year survival rate [191]. In human NSCLC cell line H460 and tumor tissue from patients with NSCLC, APS inhibited the proliferation and promoted cell apoptosis in a dose and time-dependent manner through suppressing the expression of notch1 and notch3, up-regulating the expression of p53, p21, p16, Bax and caspase-8 [192]. APS can inhibit the tumor growth of mice bearing Lewis lung carcinoma, downregulate the expression of CD44 protein, reduce collagen type IV and hyaluronic acid content, and enhance the therapeutic effect of cisplatin [193].

APS inhibit the proliferation of breast cancer cell line MDA-MB-468 through up-regulating the expressions of p53 and PTEN by regulating p53/murine double minute 2 positive and negative feedback loops [194]. In EAC breast tumor-bearing mice, APS can decrease the tumor weigh, increase immune organ indexes through activation of TLR4-mediated MyD88-dependent signaling pathway [195].

APS could prevent the side effects of chemotherapy drugs in cancer treatment, such as liver injury, cardiotoxicity and neutropenia. APS inhibit the hepatotoxicity induced by frequently-used chemical therapy agents [196]. APS could ameliorate doxorubicin-induced cardiotoxicity through restoring normal autophagic flux via regulating the AMPK/mTOR pathway, or reduce doxorubicin-induced cell apoptosis and ROS production via regulating the p38 pathway and PI3k/Akt pathway [197-199]. When neutropenia appeared in chemotherapy, APS can increase the numbers of polymorphonuclear leukocytes, and promote the differentiation and chemotactic ability of bone marrow granulocytes via the L-selectin signaling pathway [200].

### 5.2 Astragalan

Astragalan is an acidic polysaccharide isolated from *Astragalus membranaceus*.

#### 5.2.1 Anti-aging effect of Astragalan

Astragalan can offer protection against oxidative stress, by increasing antioxidant enzyme activities and decreasing peroxidative lipid levels [146,147]. In rodent models, astragalan reduces ER stress, regulates insulin/IGF-1 pathway, and thereby restores glucose homoeostasis [201,202]. Also, the regulation of insulin/IGF-1 pathway can extend lifespan [203]. In wild-type and polyglutamine (polyQ) Caenorhabditis elegans models, astragalan can reduce polyQ aggregation, alleviate neurotoxicity, and extend the adult lifespan, by regulating DAF-16 downstream genes such as scl-20, dct-5, col-84, grd-4 and spp-20 [204].

#### 5.2.2 Protection against neurodegeneration and cognitive decline by Astragalan

In an in vitro PD model of degenerate dopaminergic neurons induced by 6-Hydroxydopamine, astragalan demonstrated a protective effect against 6-OHDA neurotoxicity through alleviating the oxidative stress, inhibiting the apoptosis pathway by suppressing the proapoptotic gene egl-1 expression and increasing acetylcholinesterase activity [205].

## 6. Special monomer extract from *Astragalus membranaceus*

### 6.1 TA-65

TA-65, a single chemical entity isolated from the extract of the root of *Astragalus membranaceus*, leads to an improvement of certain health-span indicators including glucose tolerance, osteoporosis and skin fitness, without significantly increasing global cancer incidence in female mice [206].

#### 6.1.1 Anti-aging effect of TA-65

Telomeres are essential genetic elements responsible for protecting chromosomes, and short telomeres are associated with aging and many diseases [207-209]. A number of studies have shown that activating telomerase can maintain telomere length, delay aging, and reverse age tissue degeneration [210,211]. TA-65 increased telomerase activity in a telomere shortening cell model - IMR90 cells [212]. TA-65 can also lengthen the red blood cell telomere lengths and produce higher rates of feather renewal in captive zebra finches [213]. Consistent with this, TA-65 can activate telomerase to increase average telomere length in haploinsufficient mouse embryonic fibroblasts and aged mice, decrease the percentage of DNA damage and remodel the relative proportions of circulating leukocytes, reduce the percentage of cells with short telomeres and improve the structure of multiple tissues, at the same time. In old female mice, TA-65 administration for 4 months enhanced the health span, but had no impact on mean or maximum longevity [206]. A randomized, double blind, placebo controlled clinical research involving 117 relatively healthy cytomegalovirus-positive subjects aged 53-87 years old found that TA-65 can lengthen telomeres [214]. A study enrolling 7000 person-years in over a 5-year period, found that TA-65 improves markers of metabolic, bone, and cardiovascular health, with no adverse events, which suggests that TA-65 improves health and may reduce risk of morbidity and mortality [215].

#### 6.1.2 Immunoregulation by TA-65

In human bodies, TA-65 exerts positive immune remodeling effects, including significant declines in the percent of senescent cytotoxic T cells and natural killer cells, particularly in cytomegalovirus-seropositive subjects over a 1-year period relative to baseline values [216]. In cultured human CD4 and CD8 T cells from six healthy donors, it was found that TA-65 increased the telomerase activity by regulating the MAPK-specific pathway and increased proliferative activity [217].

### 6.2 TAT2

TAT2 is a single chemical entity isolated from the extract of the root of *Astragalus membranaceus*.

#### 6.2.1 Immunoregulation by TAT2

In tissue culture studies with CD8+ T cells from HIV/acquired immunodeficiency syndrome subjects, TAT2 increased replicative capacity, improved cytokine and chemokine responses to antigens, increased killing of autologous HIV-infected CD4+ cells, and retarded telomere shortening by regulating telomerase at the transcription level, probably through the regulation of the MAPK pathway [86]. And TAT2/cycloastragenol could implicate activation of c-Src, MEK, and epidermal growth factor receptor by inhibitor studies [86].

## 7. Conclusion

In this review, the current state of *Astragalus membranaceus* research involving aging and aging-related diseases is detailed and elucidated. *Astragalus membranaceus* has multiple pharmacological effects, including anti-oxidative-stress, anti-inflammatory, immuno-regulation, vascular protective effects, anti-neurodegeneration, anti-cancer and anti-aging effects via numerous signaling pathways in vital organs and systems. More attention and further studies are needed to evaluate the underlying mechanisms of *Astragalus membranaceus’* actions and its targets, in particular, reveal the mechanisms of its metabolism of absorption, distribution, transformation, and excretion. Based on the existing studies and clinical practices, *Astragalus membranaceus* has a good potential for broad application in aging and aging-related diseases.

Astragalus is usually taken in combination with other herbal supplements. When used appropriately, astragalus appears to be very safe and to have few side effects. Very high doses may suppress the immune system. Hence patients should avoid using astragalus if they are taking immune-suppressing drugs. Pregnant or nursing women should not use the astragalus root. If a person has an immune system disease, such as multiple sclerosis, lupus, rheumatoid arthritis, or another condition known as an "autoimmune disease," that person should not use the astragalus root. As with any herbal supplement, it is always necessary to check with an appropriate health care provider before taking the astragalus root.

The appropriate dose of astragalus depends on several factors, such as the user's age, health status, and several other conditions. At this time, there is not yet enough scientific information to determine an appropriate range of doses for astragalus. It is necessary to keep in mind that natural products are not always necessarily safe, and dosages can be important. It is important to follow relevant directions on product labels and consult the personal pharmacist or physician or other healthcare professional before use.
